# Catastrophic Health Expenditure and its influencing factors in Malaysia

**DOI:** 10.1186/1472-6963-12-S1-P2

**Published:** 2012-11-21

**Authors:** Sharifa Ezat WP, Azimatun Noor Aizuddin, Zakiah Zainuddin, Mohd Rizal Abd Manaf, Syed Aljunid

**Affiliations:** 1 Department of Community Health, UKM Medical Center, Kuala Lumpur, Malaysia; 2 United Nations University International Institute for Global Health, Kuala Lumpur, Malaysia

## 

Catastrophic Health Expenditure (CHE) is a term used for any expenditure for health that can pose as threat towards household's financial capacity and capability in order to maintain its subsistence needs. The World Health Organization in 2005, proposed that health expenditure be viewed as catastrophic when it is equal or exceed 40% of a household's non-subsistence income. Surveys done in 89 countries, covering 89% of the world's population suggest that annually 150 million people suffer from CHE. The ever rising healthcare cost and anticipating the nature of ill-health against extraordinary expenditure on health may lead the individual/household into CHE and poverty. In Malaysia, estimation by the Malaysia National Health Accounts, the per capita out of pocket (OOP) health expenditure is increasing, however we are highly subsidized by the government thus protecting at risk populations. With the current healthcare reform thus ensuing the proposed National Health.

Insurance and less tax financed subsidized health care, the risk of CHE is higher. In 2007, Malaysia’s total health expenditure (THE) was 4.4% of GDP; equivalent to $307 per capita and OOP making up 40.7% of THE. OOP financing exacerbate poverty and is regressive. This study plans on determining current actual and projected CHE level and its influencing factors in Malaysia. This will encourage efforts to provide higher degree of protection for low income groups against the economic impact of illness without jeopardizing the quality of services and equity of health financing. It is hoped that once the actual prevalence of CHE and its monetary level attained, these will give a strategic policy application, to reduce inequality by ensuring better access to health for all and mitigating the risk factors to the high risk populations.

